# Reference values and Z-scores for left ventricular global longitudinal strain in healthy Colombian children: an echocardiographic study

**DOI:** 10.3389/fcvm.2026.1827591

**Published:** 2026-06-05

**Authors:** Yudisay Molina-Mora, Andrés Felipe Rubio-Duarte, Yuli Salcedo-Oyola, Alicia Rincón-Rangel, Claudia C. Colmenares-Mejía, Valeria Ramirez-Jaramillo, Claudia Ximena Flórez-Rodríguez

**Affiliations:** 1 Congenital and Pediatric Heart Diseases Center, Cardiovascular Institute, Hospital Internacional de Colombia HIC—Fundación Cardiovascular de Colombia FCV—Fundación Universitaria FCV, Santander, Colombia; 2 Epidemiology and Data Analysis Unit, Hospital Internacional de Colombia HIC—Fundación Cardiovascular de Colombia FCV—Fundación Universitaria FCV, Santander, Colombia; 3 Research Unit, Hospital Internacional de Colombia HIC—Fundación Cardiovascular de Colombia FCV—Fundación Universitaria FCV, Santander, Colombia; 4 Faculty of Medicine, Pediatric Cardiology Fellowhip, Universidad El Bosque, Bogota, Colombia

**Keywords:** cardiac output, Colombia, echocardiography, global longitudinal strain, infant

## Abstract

**Background:**

Left ventricular global longitudinal strain (GLS) is a sensitive marker of systolic function that enables detection of subclinical myocardial dysfunction. Reference values are derived largely from non–Latin American populations, despite known demographic variability. We sought to establish GLS reference values in a cohort of healthy children from a Latin American country.

**Methods and results:**

This observational study included 321 healthy children older than 2 years stratified by age (2–6, 7–12, and 13–17 years). Anthropometric, physiologic, and echocardiographic variables were assessed, including left ventricular ejection fraction, velocity–time integral, and GLS. GLS distributions were analyzed by age and sex using z-scores, and correlations with echocardiographic measures were examined. Among 321 participants (median age, 10 years; interquartile range, 6–14), anthropometric measures and blood pressure increased with age, whereas heart rate declined. Left ventricular ejection fraction remained normal across groups. GLS varied significantly by age, with more negative values in younger children and progressively less negative values in adolescents. GLS demonstrated a moderate correlation with age (*r* = 0.49; *p* < 0.001) and a weak correlation with ejection fraction

**Conclusion:**

GLS in this population showed significant variation with age, becoming less negative in older children. A concordance was observed between myocardial deformation and traditional volumetric measures. These findings provide population-specific reference values and highlight the importance of considering age when interpreting GLS.

## GRAPHICAL ABSTRACT

**Figure:**
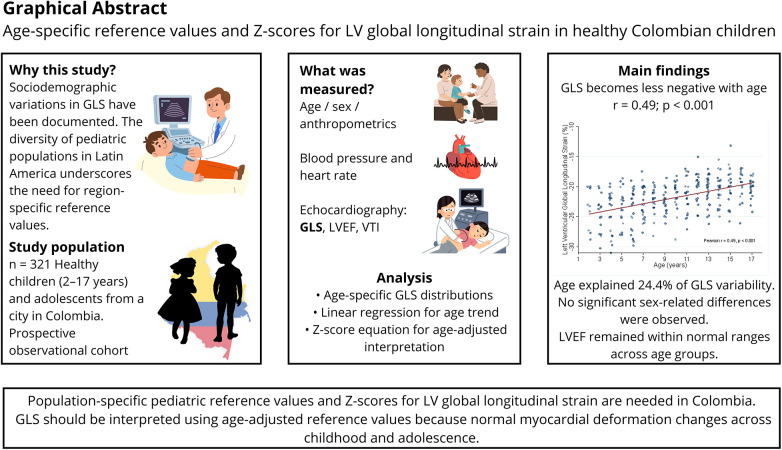

## Introduction

Left ventricular global longitudinal strain (GLS) is a key echocardiographic parameter for assessing both systolic and diastolic function and demonstrates high sensitivity for detecting subtle myocardial changes (1). This measurement is based on myocardial fiber deformation throughout the cardiac cycle and can be assessed segmentally across the entire myocardium (1). Unlike ejection fraction (EF), which is a volumetric measure, GLS shows a stronger correlation with clinical outcomes in both adult and pediatric populations, particularly during the early stages of myocardial dysfunction (2–4). However, currently available reference values (−20% to −25%) (5) are derived mainly from studies conducted in other populations, and region-specific data for Latin America—particularly Colombia—remain limited across all age groups.

Variability in GLS within established reference ranges according to factors such as race, age, and sex has been previously reported (6, 7), with less negative values observed in females, individuals of African descent, and younger children. The underlying mechanisms driving these differences remain unclear, and they have not consistently demonstrated statistical significance.

The objective of this study was to describe previously unreported reference values for GLS in a Colombian pediatric population without structural heart disease undergoing echocardiographic evaluation. In addition, age-related differences were assessed across groups ranging from 2 to 18 years.

## Methods

### Study design and participants

A retrospective observational cohort study was conducted. Cardiovascular Institute-Hospital Internacional de Colombia is a national referral center for pediatric echocardiography in eastern Colombia, where approximately 5,000 pediatric echocardiographic studies are performed annually on average (8). Patients aged 2 to 17 years referred for outpatient transthoracic echocardiography were eligible for inclusion. Indications for referral included evaluation to exclude congenital heart disease in patients with no abnormalities on physical examination.

To ensure inclusion of healthy population, all participants were required to have a normal cardiovascular physical examination, be classified as Ross functional class I or NYHA class I, and have no family history of sudden cardiac death or cardiomyopathy. The study population was enrolled between October 2023 and May 2024 at the pediatric echocardiography laboratory of our institution. As part of routine assessment, all patients underwent measurement of blood pressure, oxygen saturation, and heart rate. Exclusion criteria included prior cardiac surgery or transcatheter cardiac intervention, any structural or functional cardiac abnormality, and abnormal vital signs or weight for age at the time of evaluation.

Participants were enrolled consecutively using a convenience sampling approach. An initial sample of 150 patients meeting inclusion criteria was collected. Subsequently, the distribution of participants across age groups and sex was reviewed, and recruitment continued to ensure balanced representation across the pediatric age spectrum (2 to 17 years), targeting approximately 20 patients per year of age, equally distributed between males and females.

### Physical and echocardiographic assessment

For each participant, sex, age, weight, and height were recorded. Systolic blood pressure (SBP), diastolic blood pressure (DBP), and heart rate (HR) were measured using a PM-8000 Express multiparameter monitor (Mindray®) (9). Blood pressure was obtained with age-appropriate cuffs, with patients seated and at rest to ensure measurement accuracy. Standardized echocardiographic examinations were subsequently performed. Echocardiographic evaluation followed the *Guidelines and Standards for Performance of a Pediatric Echocardiogram* from the American Society of Echocardiography Pediatric Council (10). Pediatric transthoracic echocardiograms (TTE) were performed by two university certified echocardiography nurses, ensuring high-quality and reliable data acquisition. Two ultrasound systems were used according to service availability: Affiniti 70 with 68-3 and X5-1 probes (Philips®) and Vivid S70N with M5Sc–6s probe (General Electric®). All image analyses and measurements were performed, reviewed, and interpreted by a pediatric cardiologist with expertise in congenital heart disease serving as director of the echocardiography laboratory. During echocardiographic acquisition, velocity–time integral (VTI), left ventricular ejection fraction (LVEF) using automated function imaging (AFI), and GLS were assessed in accordance with American Society of Echocardiography recommendations, with adaptation to the institutional protocol for global and segmental cardiac function analysis (11).

### Statistical analysis

Categorical variables are presented as absolute and relative frequencies. Continuous variables are described using measures of central tendency and dispersion according to their statistical distribution. Normality of continuous variables was assessed using graphical methods and the Shapiro–Wilk test. Correlation between GLS and age was evaluated using Pearson's correlation coefficient. A two-sided *p* value < 0.05 was considered statistically significant. Age was additionally categorized into three groups: early childhood (2–6 years), middle childhood (7–12 years), and early adolescence (13–17 years).

A rank-inverse normal transformation was applied to improve model assumptions and obtain normalized GLS values (GLS_norm) (Supplementary Figure S1). A linear regression model was developed to estimate the expected mean GLS as a function of age:

GLS_norm=β_age×age+β_intercept

where *β*_age and *β*_intercept were estimated on the transformed scale. To allow clinical interpretation, coefficients were back-transformed to the original GLS scale using:

$$\begin{array}{rl} & \beta \_\mathrm{age}\_\mathrm{GLS}=\beta \_\mathrm{age}\_\mathrm{GLS}\_\mathrm{norm}\times \sigma \_\mathrm{GLS}\\ & \beta \_\mathrm{intercept}\_\mathrm{GLS}=(\beta \_\mathrm{intercept}\_\mathrm{GLS}\_\mathrm{norm}\times \sigma \_\mathrm{GLS})+\mu \_\mathrm{GLS} \end{array}$$

where *σ*_GLS and μ_GLS represent the standard deviation and mean of GLS on the original scale, respectively.

The expected GLS value for a given age was then estimated as:

GLS_expected=(β_age_GLS×age)+β_intercept_GLS

Individual GLS Z-scores were calculated to determine each participant's position relative to the reference population using:

$$z=\frac{[\mathrm{GLS}\;-\;\mathrm{GLS}\;\mathrm{expected})]\;}{\sigma \_\mathrm{residual}}$$

Residual normality was assessed using the Shapiro–Wilk test and scatterplots (Supplementary Figure S2). Potential systematic bias introduced during back-transformation was evaluated using regression models of the difference as a function of age. In addition, a spline-based regression model was explored, and linear vs. nonlinear models were compared using the likelihood ratio test (*lrtest*). The comparison yielded *χ*² = 3.58 with *p* = 0.3110, indicating that the linear model adequately described the relationship between variables without requiring nonlinear terms. In addition to the regression-based approach, age-specific expected GLS values were derived from the back-transformed model. The residual standard deviation of the model was used to calculate Z-scores and to define reference limits corresponding to Z-scores of ±2. To complement the model-based estimates, empirical percentiles (5th and 95th) were calculated from the observed GLS distribution within each age group. These results were summarized in a Supplementary Table to facilitate clinical interpretation. All analyses were performed using Stata version 15.0. The study was approved by the Institutional Research Ethics Committee of Fundación Cardiovascular de Colombia (CEI-2024–08218; July 24, 2024)

## Results

A total of 321 healthy children and adolescents were included: 121 studies were performed using an Affiniti 70 (Philips®) and 200 using a Vivid S70N (General Electric®) echocardiography system, with a median age of 10 years [interquartile range (IQR): 6–14]. Participants were distributed across three age groups: early childhood (2–6 years, *n* = 87), middle childhood (7–12 years, *n* = 125), and early adolescence (13–17 years, *n* = 109). Sex distribution was balanced in the first two groups, with a slight female predominance during adolescence (62.4%). Regarding anthropometric and physiological variables, progressive increases in weight, height, and blood pressure were observed with advancing age. Heart rate declined consistently across age groups, decreasing from 89.3 ± 10.3 beats/min in the 2–6-year group to 70.7 ± 12.7 beats/min in adolescents (Table 1).

**Table 1:** Characteristics and echocardiographic measures of the study population.

Characteristics	All cohort	Age Group (years)		
	*n* (%) (*N* = 321)	2–6 years (*n* = 87) *n* (%)	7–12 years (*n* = 125) *n* (%)	13–17 years (*n* = 109) *n* (%)
Age (years)*	10 (6–14)	4 (3–6)	10 (8–11)	15 (14–16)
Sex				
Female	181 (55.3)	45 (51.7)	65 (52.0)	68 (62.4)
Male	146 (44.6)	42 (48.3)	60 (48.0)	41 (37.6)
Anthropometric Measures				
Weight (kg)*	34 (21–50)	17 (14–20)	32 (27–40)	55 (47–60)
Height (cm)*	140 (120–159)	107 (100–115)	139 (131–147)	163 (157–169)
HR (lat/min)	78.4 (13.8)	89.3 (10.3)	77.7 (12.0)	70.7 (12.7)
SBP (mmHg)	103.0 (10.7)	95.2 (8.3)	102.5 (9.5)	109.5 (9.6)
DBP (mmHg)	65.1 (8.4)	59.4 (6.5)	64.8 (7.2)	69.9 (8.3)
MBP (mmHg)	77.7 (8.5)	71.3 (6.4)	77.4 (7.1)	83.1 (8.0)
Echocardiographic Measures				
EF (%)	64.0 (6.6)	65.7 (5.3)	64.3 (6.7)	62.3 (7.0)
VTI (cm)	23.1 (4.1)	20.8 (3.6)	23.4 (3.9)	24.7 (3.8)
GLS*	−21.8 (−23.8; −19.9)	−23 (−27.2; −21.4)	−21.4 (−23; −20)	−20 (−21.5; −18.8)

Mean (SD). *Median (Interquartile Range). HR, heart rate; SBP, systolic blood pressure; DBP, systolic blood pressure; MBP, mean blood pressure; EF, ejection fraction; VTI, velocity-time integral; GLS, global longitudinal strain.

With respect to echocardiographic measures, LVEF remained within normal ranges across all age groups, with a slight decrease during adolescence (62.3 ± 7.0%). VTI increased with age, reflecting higher stroke volume in more advanced stages of development. GS also demonstrated age-related variation. More negative values were observed in younger children [−23 (−27.2 to −21.4)], which became progressively less negative in older groups [−21.4 [−23 to −20] in 7–12 years and −20 [−21.5 to −18.8] in 13–17 years], suggesting a relative reduction in longitudinal myocardial deformation with growth.

**Figure 1:**
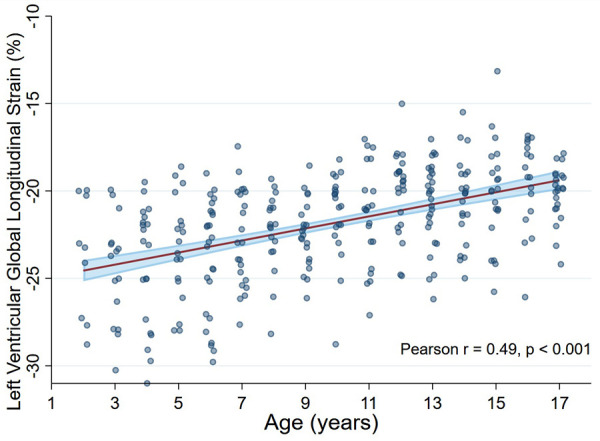
Relationship between left ventricular global longitudinal strain and age. Scatterplot showing the association between GLS and age. The solid line represents the fitted linear regression model, and dashed lines indicate the 95% confidence interval. Points correspond to individual observations. The Pearson correlation coefficient and corresponding *p* value are displayed within the panel.

**Figure 2:**
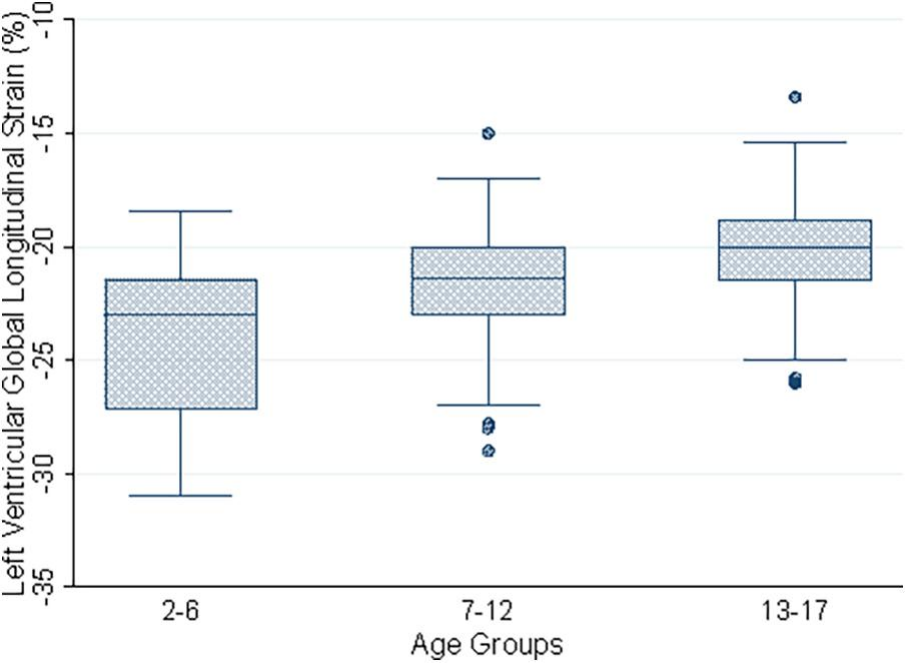
Distribution of left ventricular global longitudinal strain across age groups. Boxplots show the distribution of GLS according to age groups. The central line represents the median, and the boxes indicate the interquartile range (25th–75th percentiles). Whiskers represent the spread of the data. Individual observations are displayed as overlaid points.

Figure 1 illustrates the correlation between age and left ventricular GLS in the pediatric population studied. A positive linear trend was observed (*r* = 0.49; *p* < 0.001), indicating a progressive decrease in myocardial deformation (less negative GLS values) with increasing age. In Figure 2, a trend toward less negative GLS values with increasing age is observed, indicating a progressive reduction in left ventricular longitudinal deformation during growth. In the 2–6-year group, GLS values are more negative (median near −25), with greater dispersion and the presence of extreme values. As age advances, median values approach −20, and variability decreases, particularly in the 13–17-year group, which may reflect maturation of myocardial contractile patterns.

**Figure 3:**
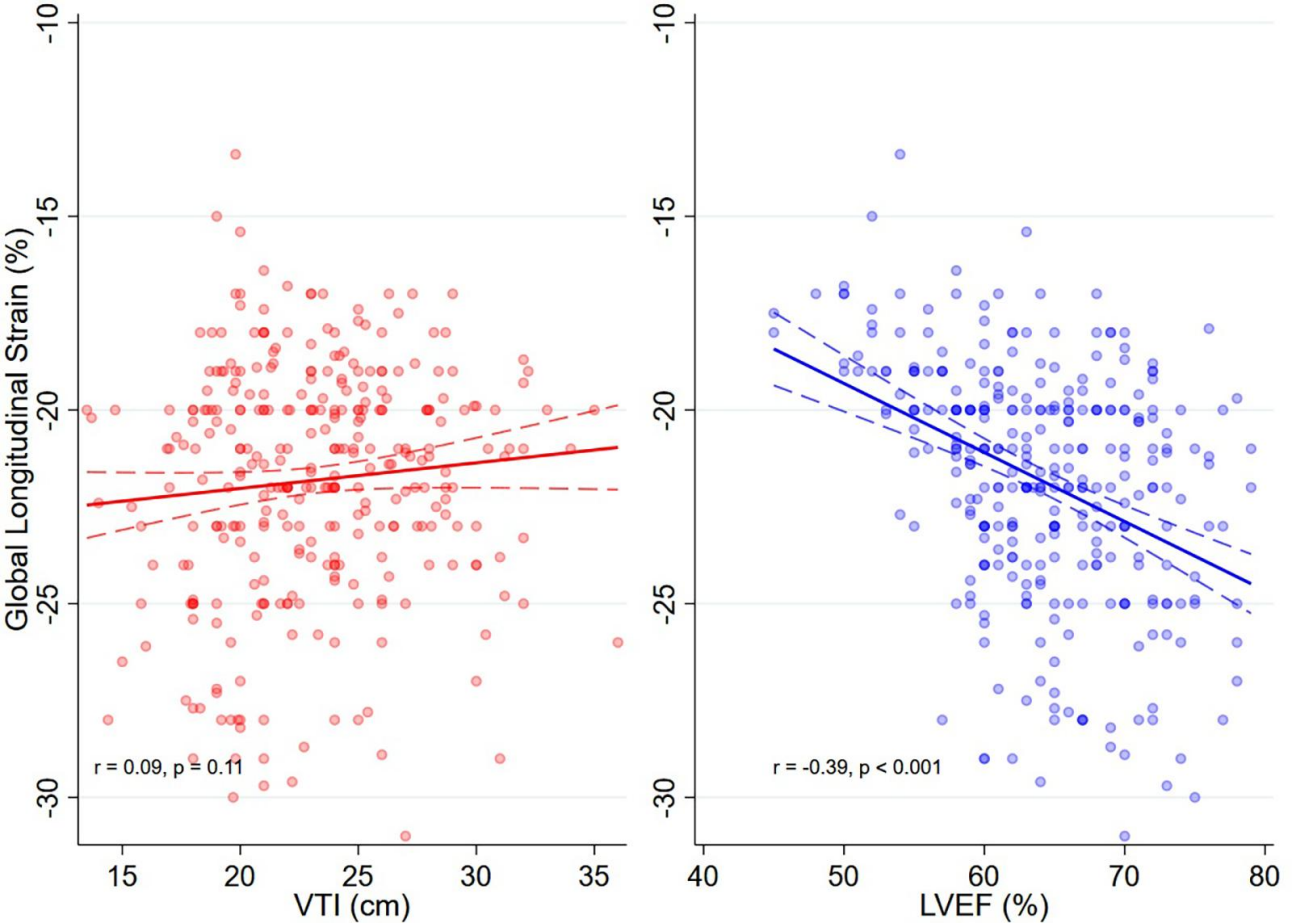
Relationship between left ventricular global longitudinal strain and echocardiographic parameters. Scatterplots illustrate the associations between GLS and velocity–time integral (VTI, left panel, red) and between GLS and left ventricular ejection fraction (LVEF, right panel, blue). Solid lines represent fitted linear regression models, and dashed lines indicate 95% confidence intervals. Points correspond to individual observations. Pearson correlation coefficients and corresponding *p* values are displayed within each panel.

Figure 3 shows the relationship between GLS and two echocardiographic variables: LVEF and VTI. A weak negative correlation was observed between GLS and LVEF (*r* = −0.39, *p* < 0.001). In contrast, GLS showed a weaker and non-significant correlation with VTI (*r* = 0.09, *p* = 0.11).

In the regression model, GLS showed a linear association with age (transformed β_age = 0.1123; 95% CI: −0.1340 to −0.0906; *p* < 0.001), indicating that increasing age is associated with less negative GLS values. Likewise, the coefficient of determination (*R*^2^ = 0.2444) suggests that approximately 24.4% of the variability in GLS can be explained by age. When additional covariates were included, only a minimal increase in explained variance was observed. The full model did not significantly improve model fit compared with the age-only model (LR *χ*² = 13.76, *p* = 0.0557), supporting the use of a parsimonious model including age alone.

Model estimates were back-transformed to the original GLS scale. Regression coefficients obtained in the transformed scale were rescaled by the standard deviation of GLS (SD = 3.0527089), while the intercept was rescaled by the same SD and subsequently adjusted by adding the mean GLS value (mean = −21.812461), thereby restoring the model to clinically interpretable units. This yielded the following age-specific equation in the original scale:

expectedGLS=0.342827×age−25.420171.

These parameters were used to estimate the expected GLS value for a given age and to construct the Z-score equation. The Z-score was calculated as the difference between the observed and expected GLS divided by the residual standard deviation of the model in the original scale (σ = 0.86299 × 3.0527089 = 2.63445725), allowing standardization of individual values relative to the age-specific reference and ensuring consistency between the statistical model and its clinical application.

The Z-score estimation can be obtained as follows:

1. Calculate expected GLS for patient age:
  expectedGLS=(0.342827×age)–25.420171
2. Obtain GLS value from the echocardiographic study
3. Calculate the difference between observed and expected GLS:
  Difference=observedGLS-expectedGLS
4. Divide this difference by the residual standard deviation in the original scale:
  Z-score=difference/2.63445725

A Z-score ≥ 2 indicates values above the expected range (less negative GLS), whereas a Z-score ≤ −2 indicates values below the expected range. Age-specific expected GLS values, along with corresponding 95% CI, Z-score–based reference limits (±2), and empirical percentiles (P5 and P95), are presented in Supplementary Table S1.

## Discussion

In this cohort of healthy Colombian children and adolescents, we established age-specific reference values and Z-scores for left ventricular GLS. The principal findings are as follows: (1) GLS demonstrated a significant age-dependent trend, becoming progressively less negative with increasing age; (2) age explained approximately one quarter of GLS variability, indicating a moderate association; (3) GLS showed a moderate inverse relationship with left ventricular ejection fraction and a weaker association with velocity–time integral; and (4) no significant sex-related differences were observed across age groups. These findings were derived from a high-volume pediatric echocardiography laboratory, a regional and national referral center for congenital heart disease in Colombia, where approximately 5,000 pediatric echocardiographic studies are performed annually under standardized acquisition and interpretation protocols.

Assessment of left ventricular systolic and diastolic function constitutes a fundamental component of echocardiographic evaluation in pediatric patients with suspected congenital or acquired heart disease. This assessment integrates multiple parameters, including ejection fraction, VTI, and more recently, global longitudinal strain, which enables reliable determination of segmental contractility through evaluation of myocardial fiber shortening. In pediatric patients, this measurement has gained increasing relevance, and its utility has been described across various congenital heart diseases associated with alterations in ventricular mechanics at different stages of disease evolution; therefore, its incorporation is considered essential in comprehensive echocardiographic evaluations (12).

During cardiac contraction, circumferential and longitudinal myocardial dimensional changes occur, which can be noninvasively assessed using echocardiography-derived GLS, allowing quantification of myocardial segmental deformation expressed as a percentage (13). Strain represents an index of regional and global deformation, defined as the fractional change in length of a myocardial segment (14), providing more detailed information on ventricular mechanics than conventional measurements and enabling detection of subclinical dysfunction, increasingly establishing GLS as a sensitive marker of ventricular dysfunction (15).

GLS as an estimator of left ventricular function has been widely used in adult populations, including its association with clinical outcomes in critically ill patients (16, 17). However, its application in pediatric practice remains limited by the scarcity of available literature. Current GLS reference ranges in pediatric populations are derived primarily from studies synthesizing reference values in healthy European and North American cohorts (18, 19). These studies demonstrate substantial variability within normal ranges according to race and geographic origin, which represents a significant limitation when applying these values to other populations such as those in Latin America. Given the pluricultural and ethnically diverse composition of this region, GLS assessment in healthy populations remains largely unexplored. Consequently, the present study provides reference values, at least for Colombian pediatric populations.

In 2025, a review of the different pediatric GLS nomograms were published, which were derived using various ultrasound platforms, with the majority of these studies conducted in Europe and North America, and a smaller number in Asia and Africa, with no population from Latin America included (20). In North American cohorts, a close relationship has been reported between GLS and conventional parameters used to estimate systolic function in pediatric populations, such as left ventricular ejection fraction and VTI (15). These findings are consistent with our results; however, they derive from relatively small cohorts in which variability related to population-specific characteristics such as sex, ethnicity, and race was not described.

Reference GLS values in healthy pediatric populations have been reported to range from −12.9% to −26.5%, with substantial heterogeneity across evaluated populations and significant differences according to software platform, left ventricular end-diastolic dimension, and age group (21). Notably, this systematic review did not include studies from Latin American populations. Other understudied populations have demonstrated differences associated with height but not age (22), whereas in African cohorts, differences were observed only by age, with no associations with sex or anthropometric parameters (23). In contrast, the Spanish cohort demonstrated inverse associations of both BSA and age with GLS values. In our cohort, variability in GLS appears to be predominantly driven by age group rather than by body surface area, weight, or height (24). Moreover, in this Colombian population, GLS showed a direct correlation with age, with no significant differences between sexes. Age explained approximately 24% of the variability in GLS in our population, indicating that additional physiological determinants contribute to myocardial deformation during growth. Similar findings have been reported in prior pediatric studies, where even multivariable models demonstrate modest explanatory capacity. For example, in the Spanish cohort, coefficients of determination as low as *R*² ≈ 0.09 have been described, with only end-diastolic volume independently associated with strain values (24). These findings further underscore that extrapolating reference values from other populations may negatively affect the diagnostic accuracy of GLS.

A strength of this study is the design with consecutive recruitment and balanced age representation, enabling construction of continuous age-based reference curves. To improve model robustness, GLS values were rank–inverse normal transformed, linear regression was performed in the transformed scale, and coefficients were subsequently back-transformed to the original GLS scale to derive clinically interpretable age-specific expected values and Z-scores. Potential non-linearity was explored using cubic splines, with likelihood ratio testing confirming that a linear model adequately described the age–GLS relationship. This approach allowed development of a single continuous reference equation applicable across the pediatric age range.

## Limitations

Several limitations should be acknowledged. First, this was a single-center study conducted in a referral laboratory in northeastern Colombia, which may limit representativeness of other regions with different ethnic compositions. Second, vendor-specific effects could not be formally evaluated because two ultrasound platforms were used and subgroup sample sizes were insufficient for stratified analyses. Although two ultrasound platforms were used in this study, prior evidence suggests that global longitudinal strain is a relatively robust parameter with good reproducibility. Reported intraobserver and interobserver intraclass correlation coefficients typically range from 0.85 to 0.95, with coefficients of variation generally below 10%. While some degree of inter-vendor variability persists, GLS has been shown to be more reproducible than other strain-derived parameters when standardized acquisition and analysis protocols are applied. These characteristics support the use of GLS in clinical and research settings despite potential variability related to imaging systems (25). Third, interobserver and intraobserver variability were not formally assessed, as reproducibility analysis was not within the scope of this study. This may limit the generalizability of GLS measurements across different operators and laboratories. All studies were reviewed and final GLS values were determined by a single experienced pediatric cardiologist to ensure consistency of measurements. Finally, although the sample size was adequate for reference modeling, residual variability indicates that factors beyond age—such as loading conditions or myocardial geometry—may also influence GLS.

The external validity of these findings is most applicable to pediatric populations with similar demographic characteristics. Multicenter studies incorporating broader geographic representation and standardized acquisition and analysis protocols will be necessary to confirm these reference ranges and assess potential ethnic or regional differences within Colombia and Latin America.

## Conclusions

In this cohort of healthy children and adolescents, GLS values varied across age groups and demonstrated significant correlations with conventional echocardiographic parameters, including LVEF and VTI. We provide the first age-specific GLS reference values and Z-scores derived from a Colombian pediatric population. Given the ethnic and demographic diversity of Latin America, population-specific reference values may improve standardized interpretation of GLS and enhance the detection of ventricular dysfunction in pediatric clinical practice. Further multicenter studies are warranted to externally validate these findings and assess their broader clinical applicability.

## Data Availability

The datasets generated and/or analyzed during the current study are not publicly available due to institutional and ethical restrictions, as they contain sensitive patient information. Requests to access these datasets should be directed the corresponding author upon reasonable request and with approval from the relevant ethics committee and the participating institution.

## References

[1] Smiseth OA Rider O Cvijic M Valkovič L Remme EW Voigt JU. Myocardial strain imaging: theory, current practice, and the future. JACC Cardiovasc Imaging. (2025) 18(3):340–81. 10.1016/J.JCMG.2024.07.011 39269417

[2] Raafs AG Boscutti A Henkens MTHM van den Broek WWA Verdonschot JAJ Weerts J. Global longitudinal strain is incremental to left ventricular ejection fraction for the prediction of outcome in optimally treated dilated cardiomyopathy patients. J Am Heart Assoc. (2022) 11(6):e024505. 10.1161/JAHA.121.024505 35253464 PMC9075270

[3] Xu H Li J Bao Z Xu C Zhang Y Liu H. Early change in global longitudinal strain is an independent predictor of left ventricular adverse remodelling in patients with right ventricular apical pacing. Heart Lung Circ. (2019) 28(12):1780–7. 10.1016/J.HLC.2018.11.004 30503810

[4] Cheung YF. Echocardiographic strain imaging: what do paediatric cardiologists need to know? Pediatric Med. (2022) 5. 10.21037/PM-21-39/COIF

[5] Yingchoncharoen T Agarwal S Popović ZB Marwick TH. Normal ranges of left ventricular strain: a meta-analysis. J Am Soc Echocardiogr. (2013) 26(2):185–91. 10.1016/j.echo.2012.10.008 23218891

[6] Russo C Jin Z Homma S Rundek T Elkind MSV Sacco RL. Race-ethnic differences in subclinical left ventricular systolic dysfunction by global longitudinal strain: a community-based cohort study. Am Heart J. (2015) 169(5):721–6. 10.1016/J.AHJ.2015.02.011 25965720 PMC4429251

[7] Singulane CC Miyoshi T Mor-Avi V Cotella JI Schreckenberg M Blankenhagen M. Age-, sex-, and race-based normal values for left ventricular circumferential strain from the world alliance societies of echocardiography study. J Am Soc Echocardiogr. (2023) 36(6):581–590.e1. 10.1016/j.echo.2022.12.018 36592875

[8] Hospital internacional de Colombia. (2026). Centro de cardiopatías congénitas y pediátricas. Available online at: https://hic.fcv.org/co/instituto-cardiovascular/servicios/cardiologia-de-congenitas-y-pediatricas/nuestro-equipo (Accessed February 4, 2026).

[9] Soma Tech Intl. Mindray PM-8000 Express—Monitores Multiparametros. Available online at: https://www.somatechnology.com/spanish/equipo-medico-usado-remanufacturado/monitores-de-signos-vitales/monitores-multiparametros/mindray-pm-8000express/ (Accessed August 12, 2024).

[10] Lai WW Geva T Shirali GS Frommelt PC Humes RA Brook MM. Guidelines and standards for performance of a pediatric echocardiogram: a report from the task force of the pediatric council of the American society of echocardiography. J Am Soc Echocardiogr. (2006) 19(12);1413–30. 10.1016/j.echo.2006.09.001 17138024

[11] Chan J Shiino K Obonyo NG Hanna J Chamberlain R Small A. Left ventricular global strain analysis by two-dimensional speckle-tracking echocardiography: the learning curve. J Am Soc Echocardiogr. (2017) 30(11):1081–90. 10.1016/J.ECHO.2017.06.010 28797723

[12] Romanowicz J Ferraro AM Harrington JK Sleeper LA Adar A Levy PT. Pediatric normal values and Z score equations for left and right ventricular strain by two-dimensional speckle-tracking echocardiography derived from a large cohort of healthy children. J Am Soc Echocardiogr. (2023) 36(3):310–23. 10.1016/j.echo.2022.11.006 36414123

[13] Amzulescu MS De Craene M Langet H Pasquet A Vancraeynest D Pouleur AC. Myocardial strain imaging: review of general principles, validation, and sources of discrepancies. Eur Heart J Cardiovasc Imaging. (2019) 20(6):605–19. 10.1093/EHJCI/JEZ041 30903139 PMC6529912

[14] Cañón-Montañez W Santos ÁBS Foppa M. Strain longitudinal global: un parámetro útil para evaluar disfunción ventricular izquierda subclínica en el síndrome metabólico. Rev Colomb Cardiol. (2016) 23(2):112–9. 10.1016/J.RCCAR.2015.10.008

[15] Singh GK Cupps B Pasque M Woodard PK Holland MR Ludomirsky A. Accuracy and reproducibility of strain by speckle tracking in pediatric subjects with normal heart and single ventricular physiology: a two-dimensional speckle-tracking echocardiography and magnetic resonance imaging correlative study. J Am Soc Echocardiogr. (2010) 23(11):1143–52. 10.1016/j.echo.2010.08.010 20850945 PMC3755612

[16] Buggey J Alenezi F Yoon HJ Phelan M DeVore AD Khouri MG. Left ventricular global longitudinal strain in patients with heart failure with preserved ejection fraction: outcomes following an acute heart failure hospitalization. ESC Heart Fail. (2017) 4(4):432–9. 10.1002/EHF2.12159 29154416 PMC5695196

[17] Brann A Miller J Eshraghian E Park JJ Greenberg B. Global longitudinal strain predicts clinical outcomes in patients with heart failure with preserved ejection fraction. Eur J Heart Fail. (2023) 25(10):1755–65. 10.1002/EJHF.2947 37369633

[18] Levy PT Machefsky A Sanchez AA Patel MD Rogal S Fowler S. Reference ranges of left ventricular strain measures by two-dimensional speckle-tracking echocardiography in children: a systematic review and meta-analysis. J Am Soc Echocardiogr. (2016) 29(3):209–225.e6. 10.1016/j.echo.2015.11.016 26747685 PMC4779733

[19] Koopman LP Rebel B Gnanam D Menting ME Helbing WA Boersma E. Reference values for two-dimensional myocardial strain echocardiography of the left ventricle in healthy children. Cardiol Young. (2019) 29(3):325–37. 10.1017/S1047951118002378 30777588

[20] Cantinotti M Capponi G Marchese P Franchi E Santoro G Assanta N. Normal values for speckle-tracking echocardiography in children: a review, update, and guide for clinical use of speckle-tracking echocardiography in pediatric patients. J Clin Med. (2025) 14(4):1090. 10.3390/JCM14041090 40004621 PMC11856153

[21] Jashari H Rydberg A Ibrahimi P Bajraktari G Kryeziu L Jashari F. Normal ranges of left ventricular strain in children: a meta-analysis. Cardiovasc Ultrasound. (2015) 13(1):37. 10.1186/S12947-015-0029-0 26250696 PMC4528396

[22] Voges I Negwer I Caliebe A Boroni Grazioli S Daubeney PEF Uebing A. Myocardial deformation in the pediatric age group: normal values for strain and strain rate using 2D magnetic resonance feature tracking. J Magn Reson Imaging. (2022) 56(5):1382–92. 10.1002/JMRI.28073 35072310

[23] Kotby AA Ebrahim SOS Al-Fahham MM. Reference centiles for left ventricular longitudinal global and regional systolic strain by automated functional imaging in healthy Egyptian children. Cardiol Young. (2023) 33(1):26–34. 10.1017/S1047951122000129 35241202

[24] Aristizábal-Duque CH Cabeza JF Sánchez IMB Ortega MD Martinez PA Romero-Saldaña M. The assessment of myocardial longitudinal strain in a paediatric Spanish population using a new software analysis. J Clin Med. (2022) 11(12):3272. 10.3390/JCM11123272 35743343 PMC9224625

[25] Patrianakos AP Zacharaki AA Kalogerakis A Solidakis G Parthenakis FI Vardas PE. Two-dimensional global and segmental longitudinal strain: are the results from software in different high-end ultrasound systems comparable? Echo Res Pract. (2015) 2(1):29–39. 10.1530/ERP-14-0070 26693313 PMC4676462

