# Supplementary material for: Reference values and Z-scores for left ventricular global longitudinal strain in healthy Colombian children: an echocardiographic study
Source: Front Cardiovasc Med. 2026 Jun 5;13:1827591. doi: 10.3389/fcvm.2026.1827591 (PMC13279712; doi:10.3389/fcvm.2026.1827591)
Supplement: Supplementary file 1 [file Datasheet1.docx]

**Rank–Inverse Normal Transformation**

To satisfy linear regression assumptions, global strain (GLS) values were transformed using a rank–inverse normal transformation (RINT). Briefly, GLS values were ranked and mapped onto the standard normal distribution according to:

$$G{LS}_{norm}=\Phi^{-1}\left( \frac{rank(GLS)-0.5}{N} \right),$$

where $\Phi^{-1}$denotes the inverse cumulative distribution function of the standard normal distribution and $N$is the sample size.

**Figure S1. Histogram of normalized LV global longitudinal strain**

**Linear Regression Modeling**

A linear regression model was fitted using the transformed GLS values:

$$G{LS}_{norm}=\beta_{age}^{norm}\cdot age+\beta_{0}^{norm}.$$

Model assumptions were evaluated through inspection of residual distributions and Shapiro–Wilk testing.

**Figure S2. Residual of Linear regression of normalized GLS and age**

**Back-transformation to the Original GLS Scale**

To obtain clinically interpretable reference values, regression coefficients estimated in the transformed scale were back-transformed to the original GLS scale using the mean ($\mu_{GLS}$) and standard deviation ($\sigma_{GLS}$) of GLS:

$$\beta_{age}^{GLS}=\beta_{age}^{norm}\cdot\sigma_{GLS},$$

$$\beta_{0}^{GLS}=\beta_{0}^{norm}\cdot\sigma_{GLS}+\mu_{GLS}.$$

Age-specific expected GLS values were then computed as:

$$G{LS}_{expected}=\beta_{age}^{GLS}\cdot age+\beta_{0}^{GLS}.$$

**Assessment of Non-linearity**

To explore potential non-linear associations between age and GLS, cubic spline regression models with four knots were fitted in the transformed scale. Model fit between linear and spline models was compared using likelihood ratio testing. As no significant improvement was observed with spline terms, the linear model was retained.

**Supplementary Table 1. Age-specific expected GLS values, 95% confidence intervals, Z-score–based reference limits (±2), and empirical percentiles (P5 and P95).**

| **Age** | **Expected GLS (%)** | **95% CI** | | **Z = −2** | **Z = +2** | **P5** | **P95** |
| --- | --- | --- | --- | --- | --- | --- | --- |
| 2 | -24,73 | -25,33 | -24,13 | -30,05 | -19,42 | -28,7 | -19,9 |
| 3 | -24,39 | -24,93 | -23,85 | -29,7 | -19,08 | -30 | -20 |
| 4 | -24,05 | -24,54 | -23,56 | -29,36 | -18,74 | -30,3 | -19,75 |
| 5 | -23,71 | -24,14 | -23,27 | -29,02 | -18,39 | -28 | -18,4 |
| 6 | -23,36 | -23,75 | -22,97 | -28,68 | -18,05 | -29 | -20 |
| 7 | -23,02 | -23,37 | -22,67 | -28,33 | -17,71 | -26 | -19 |
| 8 | -22,68 | -22,99 | -22,36 | -27,99 | -17,37 | -28 | -19,2 |
| 9 | -22,33 | -22,63 | -22,04 | -27,65 | -17,02 | -25 | -20 |
| 10 | -21,99 | -22,28 | -21,7 | -27,3 | -16,68 | -27 | -18,65 |
| 11 | -21,65 | -21,95 | -21,35 | -26,96 | -16,34 | -25,3 | -17,4 |
| 12 | -21,31 | -21,62 | -20,99 | -26,62 | -15,99 | -25 | -17,9 |
| 13 | -20,96 | -21,31 | -20,61 | -26,28 | -15,65 | -25 | -18 |
| 14 | -20,62 | -21,01 | -20,23 | -25,93 | -15,31 | -24 | -17 |
| 15 | -20,28 | -20,72 | -19,84 | -25,59 | -14,97 | -24 | -16,4 |
| 16 | -19,93 | -20,43 | -19,44 | -25,25 | -14,62 | -26 | -16,8 |
| 17 | -19,59 | -20,14 | -19,04 | -24,9 | -14,28 | -23 | -18 |

Note: Expected GLS values were derived from the regression model. Reference limits correspond to Z-scores of ±2 based on the residual standard deviation. Percentiles (P5 and P95) were calculated from the observed data within each age group.
